# Pediatric Dentists’ Practice Patterns in the Screening, Diagnosis, and Management of Temporomandibular Disorders

**DOI:** 10.3390/children11101168

**Published:** 2024-09-26

**Authors:** Caroline M. Sawicki, Linda Sangalli

**Affiliations:** 1 Department of Pediatric Dentistry and Dental Public Health, University of North Carolina, Chapel Hill, NC 27599, USA; 2 College of Dental Medicine—Illinois, Midwestern University, Downers Grove, IL 60515, USA; lsanga@midwestern.edu

**Keywords:** temporomandibular disorders, orofacial pain, pain management, pediatric dentistry, provider training

## Abstract

Background/Objectives: Pain associated with temporomandibular disorders (TMDs) is the main non-odontogenic cause of orofacial pain among youth. Pediatric dentists are often the first healthcare practitioners to perform a comprehensive oral examination in children, which should include evaluation of the temporomandibular joint, masticatory muscles, and surrounding structures for signs and/or symptoms of TMD. This study assessed pediatric dentists’ practice patterns in the screening, diagnosis, and management of TMD. Methods: A 19-item online survey was used to assess pediatric dentists’ clinical practice patterns and perceived knowledge and comfort levels in the screening, diagnosis, and management of TMD. The survey was distributed in July 2024, with a one-month window open for the study. Confidence levels and perceived knowledge were compared according to years from graduation and frequency of TMD screening assessment with ANOVA or independent *t*-test, as appropriate. Open-ended items were analyzed thematically. Results: A total of 206 pediatric dentists and pediatric dentistry residents participated in this study. A share of 72.8% of respondents reported that up to 25% of their patients per week present with signs and symptoms of TMD. About one-fifth of pediatric dental providers never complete a screening history for TMD (22.1%) or a clinical assessment of TMD-related structures (21.6%). There was overall low self-perceived knowledge and confidence among pediatric dentists regarding the diagnosis, screening, and management of TMD in children and adolescents, regardless of the number of years from graduation. Respondents who reported seeing up to 50% of TMD patients reported significantly more knowledge and confidence in diagnosing pediatric TMD than those who encountered fewer TMD patients. An overwhelming majority (81.6%) indicated a need for continuing education courses and training focused on the management of TMD in pediatric patients. Conclusions: Providers reported low confidence and self-perceived knowledge about TMD in pediatric populations, a high rate of referral of patients with TMD, and a particular interest for professional development opportunities focused on management of TMD.

## 1. Introduction

Pediatric orofacial pain can have significant negative psychological and physical consequences in children and adolescents [1], especially when chronic, orofacial pain in children and adolescents may be associated with emotional stress, depression, functional avoidance, and poor sleep quality [1]. The most common chronic pediatric orofacial pain complaint is temporomandibular disorders (TMDs), with prevalence rates ranging from 4.2 to 68% [2,3,4,5,6]. This wide variation in prevalence may be due to methodological differences among studies, such as lack of standardization in the diagnosis of TMD, or related to participant selections that do not represent the general population [7,8]. Nevertheless, it is clear that children and adolescents with TMD, especially TMD-related pain, comprise a substantial group with an obvious treatment need [9].

A significant barrier to the appropriate management of TMD and TMD-related pain in children and adolescents lies in the difficulty of diagnosing TMD in these populations. In an effort to address these challenges, the adult version of the Diagnostic Criteria for TMD (DC/TMD) has been adapted for use in children and adolescents [9,10]. Furthermore, the American Academy of Pediatric Dentistry (AAPD) provides updated clinical practice guidelines to assist pediatric dentists in the diagnosis and management of TMD [11]. Despite the availability of these resources, it is unclear whether pediatric dental providers are performing regular screening and diagnosis of TMD in their practice. Since early diagnosis can influence therapeutic success, it is critical for pediatric dentists to identify children and adolescents with signs and symptoms of TMD in its early stages, in order to provide a diagnosis and recommend appropriate management strategies. Related to this, several studies have suggested a relationship between psychosocial factors and TMD in adolescent patients [12,13]. Therefore, pediatric dentists may also play an important role in intercepting potential emotional issues underlying or exacerbating TMD and TMD-related pain and referring patients to appropriate providers.

According to the AAPD, all comprehensive dental examinations in pediatric patients should consist of history taking and clinical examination of the temporomandibular joint (TMJ) and surrounding area [11]. Although it is expected that pediatric dentists are aware of significant signs and symptoms of TMD in children and adolescents, the Commission on Dental Accreditation (CODA) does not require specific education in the screening, assessment, diagnosis, and/or management of TMD in pediatric dentistry postgraduate curricula. Therefore, it is possible that pediatric dentistry residency programs are inadequately preparing graduates to assess for TMD, thus potentially leading to under-diagnosis and under-treatment of TMD in pediatric populations.

The purpose of the current study was to comprehensively assess pediatric dental providers’ clinical practice patterns, in addition to confidence levels and perceived knowledge, in the assessment, diagnosis, and management of TMD in children and adolescents. This study also aimed to identify priority areas for improving the screening and management of pediatric TMD and gather input regarding interest in educational resources pertaining to TMD. The hypothesis was that the majority (>50%) of pediatric dentists do not routinely assess for TMD signs and symptoms in their practice, with related low levels of perceived knowledge and comfortability in the screening, diagnosis, and management of TMD in pediatric patients. Understanding current clinical practice patterns among pediatric dentists and gaps in knowledge pertaining to the screening, diagnosis, and management of TMD is fundamental to improving the management of TMD and TMD-related pain in pediatric dental patients.

## 2. Materials and Methods

### 2.1. Study Design

This cross-sectional, survey study was reviewed by the University of North Carolina Institutional Review Board (IRB) and deemed exempt (24-0501, 24 March 2024). An anonymous online questionnaire was distributed via Qualtrics to all members of the AAPD. Eligible participants needed to be completing or have already completed specialized training in pediatric dentistry. Electronic informed consent was obtained from respondents prior to participation. In accordance with local IRB regulations, participants were not obligated to respond to all survey items.

### 2.2. Survey Assessment Tool

The anonymous survey (Supplementary Materials) was co-developed by a pediatric dentist (C.M.S.) and orofacial pain specialist (L.S.) who are specialized in pediatric orofacial pain and TMD management. Survey content was also reviewed by external content experts to ensure relevance and appropriateness of questions, as well as readability and accessibility. The final survey consisted of 19 items across 3 sections. Responses to all sections were optional. Section 1 assessed respondents’ perceived knowledge and comfort levels in the assessment, diagnosis, and management of TMD in pediatric patients. Section 2 evaluated the frequency by which respondents routinely conduct a screening history and perform a clinical assessment of the masticatory muscles and TMJ function in practice. Finally, Section 3 gathered information regarding respondents’ current clinical encounters of TMD and recommended management strategies for TMD, in addition to interest in obtaining educational resources pertaining to the diagnosis and management of TMD in children and adolescents. Opportunities to elaborate on close-ended responses were provided throughout via optional open text boxes, and the last item of the survey was an open text box with an invitation to “share any other thoughts or feedback related to managing TMD in pediatric dental patients”. The survey also assessed sociodemographic and professional characteristics.

### 2.3. Data Analysis

Frequencies and proportions were calculated for categorical items, while means and standard deviations were calculated for continuous items. Confidence levels, perceived knowledge, and frequency of screening assessment were compared across respondents according to the number of years from graduation from residency with analysis of variance (ANOVA), and according to the percentage of TMD patients routinely encountered in clinical practice. Significant ANOVAs were run with post hoc Tukey tests. Confidence levels and perceived knowledge about TMD were then compared across the respondents according to the frequency of screening assessment. Specifically, the respondents were assigned to two groups: those who reported to “always” or “most of the time” conduct a screening history and clinical assessment were assigned a value of 1; those who reported to “never” or “sometimes” conduct history and clinical assessment were assigned a value of 0. Next, the two groups were compared with independent *t* tests in confidence levels and perceived knowledge. Effect sizes were computed with eta-squared, Cohen’s d, and Cramer’s V, as appropriate. Finally, open-ended text responses related to areas for improving education on TMD and interest in educational resources were first reviewed to identify overlapping with close-ended response options and most common patterns (themes) were summarized through thematic analysis. The statistical significance level was set at α = 0.05. Statistical analyses were conducted with SPSS (IBM SPSS, v27, IBM Corp., Armonk, NY, USA).

## 3. Results

### 3.1. Sample Characteristics

A total of 208 responses were obtained. Two of the answers submitted were excluded from the final analysis as they were provided by individuals who were neither pediatric dentists nor residents of postgraduate pediatric dentistry programs. As such, the analysis was completed on the data provided by 206 pediatric dentists or residents of postgraduate pediatric dentistry programs (65.5% females). Based on post hoc power analysis, with a significant level of 0.05 and power set at 80%, a sample size of 206 allows for the detection of a difference of approximately 10% in proportions between pediatric dentists who routinely assess TMD and those who do not. Thus, this level of power is sufficient for the primary aim of the current study, which is to detect clinically meaningful differences in practice patterns.

Table 1 shows demographic characteristics of the respondents. The most common additional specialty training was the General Practice Residency (GPR) program obtained by 16.5% of the respondents, followed by Advanced Education in General Dentistry (AEGD, 6.3%), and Orthodontics and Dentofacial Orthopedics (5.8%). Almost half of the respondents received additional training relevant to TMD through continuing education courses (49.0%), while 43.7% of them did not receive any additional training on TMD.

### 3.2. Frequency of Management of Patients with TMD Signs and Symptoms

Most of the respondents (72.8%) reported that up to 25% of their patients per week present with TMD signs and symptoms, while as high as 19.4% reported to not have any patients presenting with TMD signs and symptoms. While 6.3% of the respondents admitted to not routinely assessing for TMD in their patients, only 1.5% of the respondents indicated having up to 50% of patients with TMD signs and symptoms.

**Figure 1:**
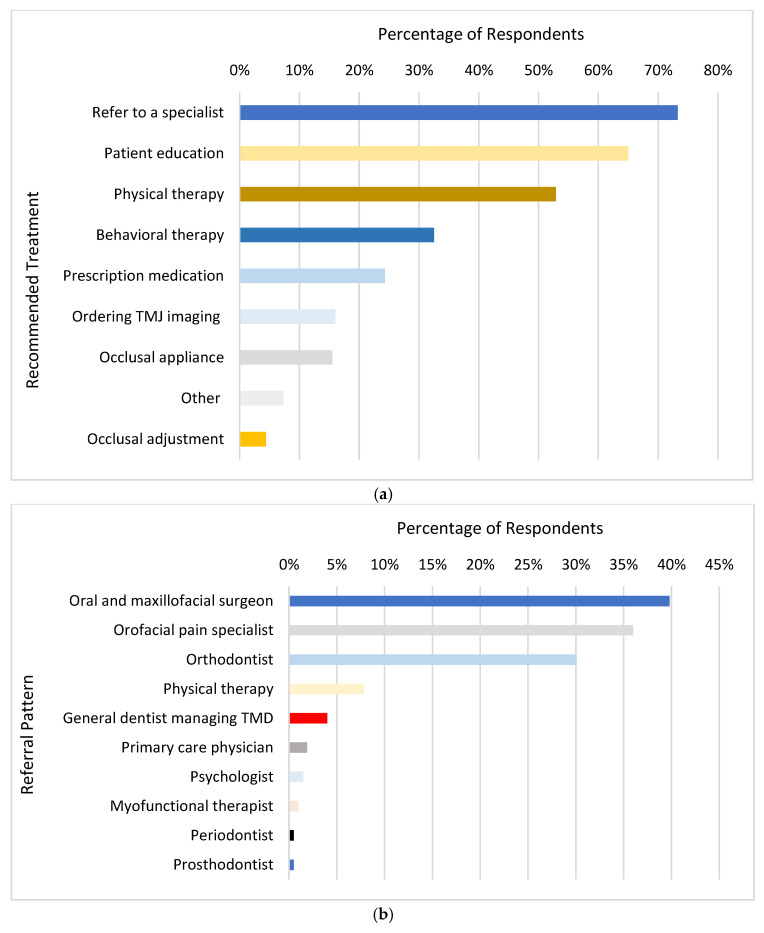
Pediatric dental providers’ management of patients with TMD. (**a**) represents respondents’ recommended treatments for pediatric patients with TMD. (**b**) represents respondents’ referral patterns for pediatric patients with TMD.

The most common age group of patients presenting with TMD signs and symptoms consisted of adolescents between 15 and 17 years (68.4%), followed by adolescents between 12 and 14 years (47.6%). As high as 73.3% of the pediatric dentists reported referring the patients to a specialist, when presenting with TMD signs and symptoms. The most common proposed treatments for TMD in pediatric patients were, in ascending order, patient education (65.0%), referral to physical therapy for jaw exercises and massage (52.9%), behavioral therapy (32.5%), and prescription medications (24.3%, Figure 1a). Pediatric patients presenting with TMD were most referred to oral and maxillofacial surgeons (39.8%), followed by orofacial pain specialists (36.0%), and orthodontists (7.8%, Figure 1b). Pediatric patients with TMD most commonly presented with comorbid conditions, including headache/migraine (57.8%), anxiety symptoms (54.4%), sleep disorder (35.0%), depression (22.8%), behavioral/developmental disorders (24.8%), and allergies (10.2%). The remaining reported comorbid conditions (arthritis, irritable bowel syndrome, bruxism, immunodeficiencies) had a frequency of less than 5.0%. Only 2.0% of the respondents indicated that there were no other comorbid conditions associated with TMD.

### 3.3. Self-Perceived Knowledge and Confidence Levels about TMD in Pediatric Populations

There was an overall low self-perceived knowledge and confidence among pediatric dentists about TMD-related aspects. The item with the highest confidence level was the screening of TMD in pediatric populations (50.3 ± 31.7), followed by general knowledge about TMD (43.6 ± 27.0) and confidence in diagnosing TMD (41.3 ± 29.8). The item which achieved the lowest score in confidence level was the management of TMD (30.4 ± 27.9).

The levels of self-perceived knowledge and confidence about TMD in pediatric populations did not significantly differ according to years from graduation (Table 2) nor according to the primary clinical working setting (all *p*’s > 0.05).

**Figure 2:**
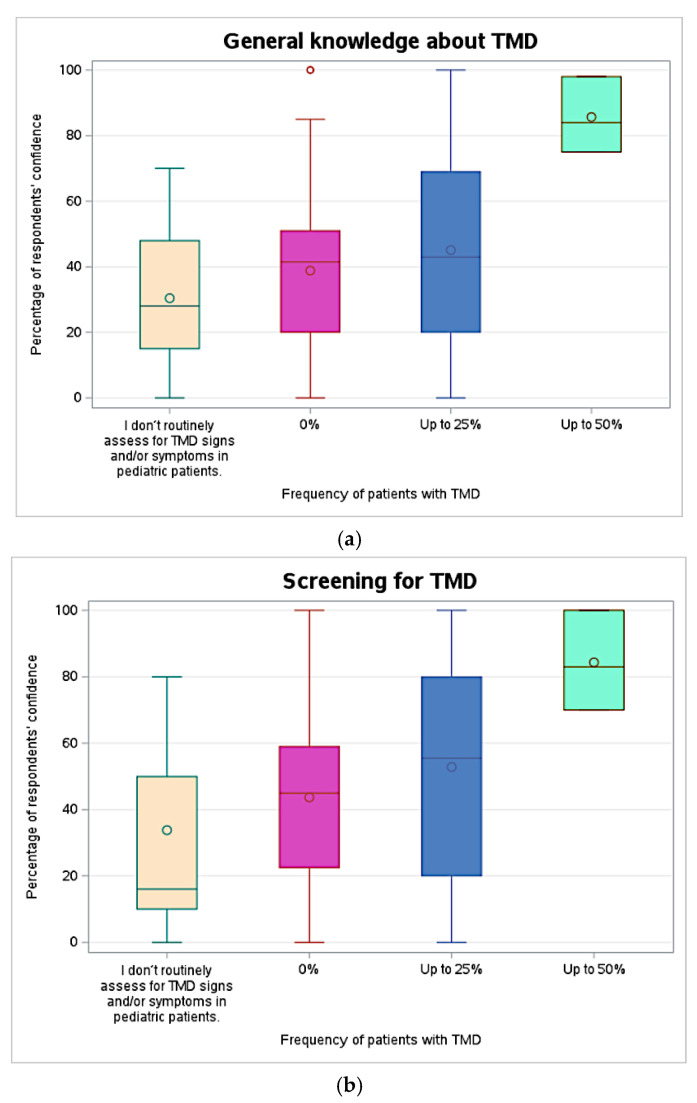

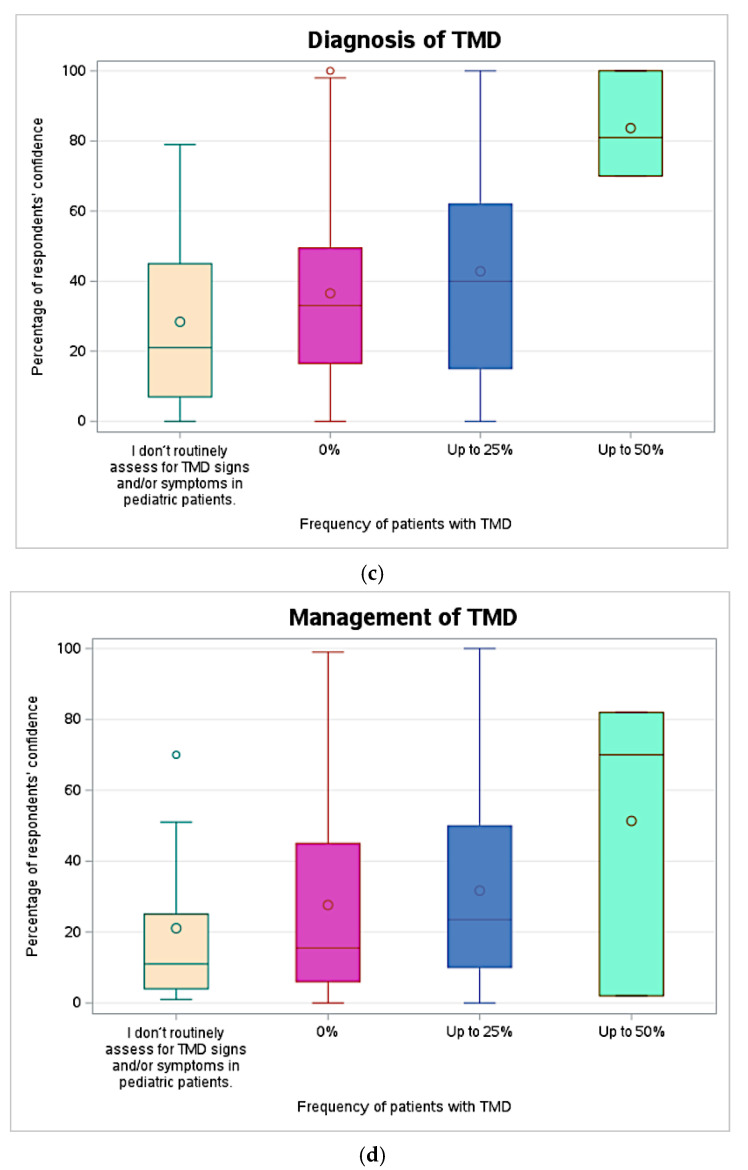

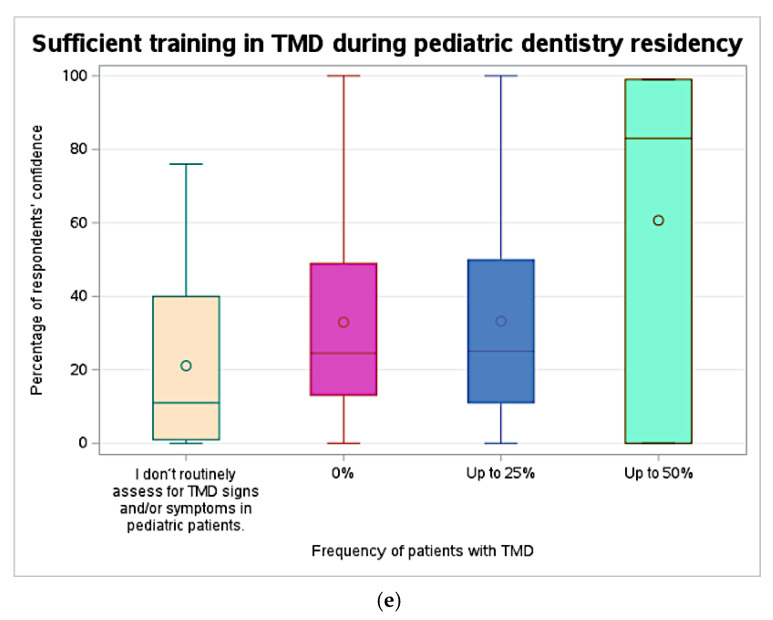
Respondents’ self-reported confidence level and self-perceived knowledge about TMD in pediatric patients according to respondents’ self-reported percentage of TMD patients seen in clinical practice. (**a**) represents differences in self-perceived general knowledge about TMD; (**b**) represents differences in self-perceived confidence in screening for TMD; (**c**) represents differences in self-perceived confidence in diagnosis of TMD; (**d**) represents differences in self-perceived confidence in management of TMD; and (**e**) represents differences in self-perceived training in TMD during pediatric dentistry residency.

However, confidence levels in the diagnosis of TMD (*p* = 0.018, effect size = 0.05) and self-perceived knowledge about TMD (*p* = 0.006, effect size = 0.06) in pediatric populations differed according to the percentage of TMD patients routinely seen in clinical practice (Figure 2). Specifically, the pediatric dentists who reported seeing up to 50% of patients with TMD signs and symptoms considered themselves significantly more knowledgeable about TMD in pediatric populations than those who reported seeing up to 25% of TMD patients (85.7 ± 11.6 vs. 45.1 ± 27.1, *p* = 0.045), those who reported seeing 0% of TMD patients (85.7 ± 11.6 vs. 38.9 ± 25.7, *p* = 0.018), and those who reported not routinely screening for TMD (85.7 ± 11.6 vs. 30.4 ± 21.8, *p* = 0.007, Figure 2a). Similarly, the pediatric dentists who reported seeing up to 50% of patients with TMD signs and symptoms scored significantly higher in confidence level in diagnosis of TMD than those who reported seeing 0% of TMD patients (83.7 ± 15.2 vs. 36.5 ± 27.2, *p* = 0.038) and those who reported not routinely screening for TMD (83.7 ± 15.2 vs. 28.4 ± 26.0, *p* = 0.018, Figure 2c). These differences occurred despite no significant differences observed in how sufficient their pediatric residency training was perceived on TMD (Figure 2e). Conversely, no significant differences were observed among respondents in confidence levels regarding screening (Figure 2b) and management of TMD (Figure 2d).

Interestingly, compared to those who reported never/sometimes conducting TMD screening history and clinical assessment in their patients, those who reported always/most of the time doing it scored significantly higher in self-perceived knowledge and confidence levels in management, diagnosis, and screening (all *p* < 0.05, Table 3). They also reported significantly greater training on TMD during the pediatric dentistry residency compared to those who never or sometimes conducted TMD screening assessment (all *p* < 0.05, Table 3).

**Figure 3:**
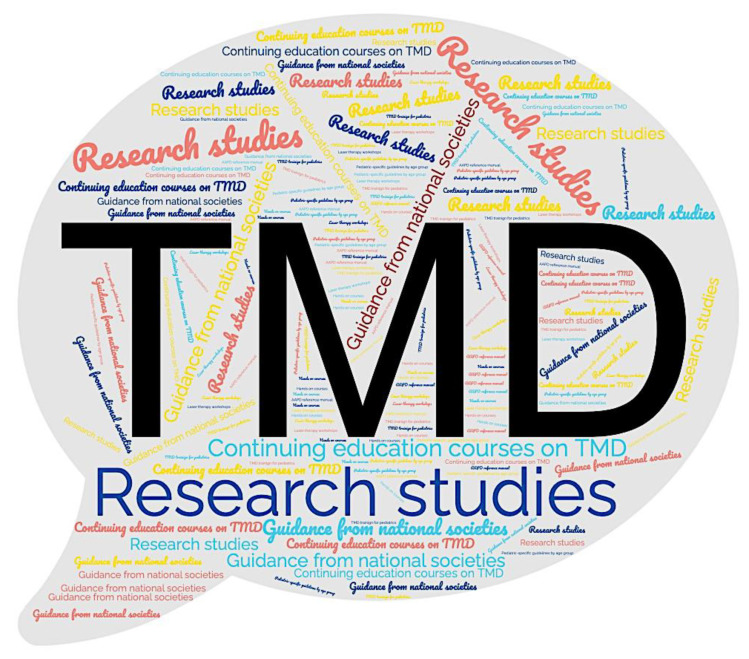
Educational resources endorsed by pediatric dental providers. The word cloud visually represents the frequency of words utilized to describe helpful educational resources for pediatric dentists working with patients with TMD signs and symptoms. The size of each word reflects the frequency of educational resources indicated by the respondents, with larger words appearing more often. This thematic analysis derives from the responses of N = 206 respondents to the question “Which educational resource would be most helpful for you as a pediatric dentist working with pediatric patients presenting with TMD signs and symptoms?”.

Figure 3 displays the most helpful educational resources when encountering pediatric patients with TMD, as identified by the respondents. The vast majority indicated the need of continuing education courses and training focused on the management of TMD (81.6%), followed by guidelines from national societies and research studies (76.7%).

## 4. Discussion

It is known that many children diagnosed with TMD will experience adaptive physiological changes during craniofacial growth and development [14]; however, failure to diagnose TMD in its early stages may cause chronification of TMD and significant pain-related symptoms [15,16]. Therefore, it is critical for pediatric dentists to be competent in the diagnosis and management of initial TMD signs and/or symptoms in pediatric patients. The current study aimed to evaluate pediatric dentists’ practice patterns in the screening, diagnosis, and management of TMD. Our findings indicated that about one-fifth of pediatric dental providers never complete a screening history for TMD or a clinical assessment of TMD-related structures. However, almost three-quarters of respondents reported that they see up to 25% of patients per week presenting with TMD signs and symptoms. Overall, pediatric dental providers reported low self-perceived knowledge and confidence in TMD-related aspects, with TMD management receiving the lowest score in confidence. The vast majority of pediatric dental providers endorsed more continuing education courses and professional training devoted to the management of TMD in pediatric populations.

The AAPD recognizes that TMD is a major cause of non-odontogenic pain in the orofacial region [17,18], and that disorders of the TMJ, masticatory muscles, and associated structures occur in infants, children, adolescents, and individuals with special health care needs [11]. This is in accordance with the current study’s findings, with the majority (72.8%) of pediatric dental providers indicating that they see up to 25% of patients per week with TMD signs and symptoms. In fact, 1.5% of respondents reported having up to 50% of patients per week presenting with TMD signs and symptoms. These findings emphasize the importance of pediatric dental providers performing routine screening of patients for TMD in order to be able to make an accurate diagnosis and develop a comprehensive preventive and therapeutic program for each patient. Failure to recognize and diagnose TMD signs and symptoms at initial onset can have a significant longstanding impact; for instance, adolescents with self-reported TMD pain have a three-fold higher risk of having recurring TMD pain as young adults [19]. Therefore, early diagnosis and intervention are crucial for alleviating symptoms, minimizing risk of complications, and fostering long-term patient well-being.

However, many pediatric dental providers may feel hesitant to screen or diagnose patients for TMD due to limited exposure of TMD education during their training. In support of this, current findings demonstrate an overall low self-perceived knowledge and confidence levels about TMD-related aspects among respondents. Although levels of self-perceived knowledge and self-reported confidence did not differ according to number of years from graduation, they did differ according to the percentage of TMD patients that respondents reported they routinely see in clinical practice. For instance, respondents who reported always/most of the time conducting TMD history screening and clinical assessment, also scored significantly higher in self-perceived knowledge and confidence levels in screening, diagnosis, and management of TMD. Clinical experience and patient-related variables have been shown to influence medical and dental providers’ self confidence in clinical practice patterns [20,21]. Therefore, sufficient knowledge and confidence of TMD-related aspects may be imperative for the practice of pediatric dental providers who encounter patients with TMD signs and symptoms more routinely. Similarly, it may be posed that clinical exposure to certain conditions may reinforce and enhance self-perceived knowledge and confidence levels [22]. As such, greater clinical exposure to pediatric TMD patients during specialty training may be critical to increase the pediatric dentists’ confidence. As this was a cross-sectional study in nature, we can only speculate on the direction of this association.

Relatedly, four-fifths of the sample strongly endorsed the need for continuing education courses and training focused on the management of TMD, suggesting that TMD education is a high priority area amongst pediatric dental providers. Although the AAPD provides clinical guidance to assist pediatric dental providers in the recognition and diagnosis of TMD [11], and to identify possible treatment options, a significant gap exists in clinical application of these recommendations. In support of this, over three-quarters of the sample endorsed more guidelines from national societies, such as AAPD, suggesting that current resources for TMD are not sufficient for pediatric dental providers. Follow-up studies aiming to understand provider perception of what needs to be improved in their TMD management practices, as well as provider-perceived comfort and competency using existing resources, could inform tailored knowledge mobilization and training efforts.

Findings from the current study highlight a significant gap in the clinical care of pediatric dental patients, namely, screening for TMJ health, diagnosing TMD, and facilitating treatment of TMD. Given the increasing prevalence and substantial impact of TMD in children and adolescents, future research is necessary to improve training and clinical practice in TMD-related aspects for pediatric dental providers. Furthermore, current findings suggest that gaps in TMD education may exist amongst pediatric dentistry residency programs, which warrant further investigation. For example, identification of specific educational gaps in postgraduate pediatric dentistry curricula have the potential to influence educational policies, accreditation standards, and informed allocation of resources. Finally, the study’s results imply that current clinical guidelines and recommendations may not be sufficient for pediatric dental providers; future studies should examine how these resources should be revised to address the needs of pediatric dental providers.

The current findings must be interpreted in the context of this study’s limitations. The main limitation is the low response rate, despite the authors’ efforts. A similar response rate was obtained by a study assessing pediatric dental providers’ perspectives on pain management practices and needs in pediatric dentistry [23]. The low response rate can be attributed to several factors, including limited awareness of the topic amongst pediatric dental providers or the belief that TMD management primarily falls within the domain of orofacial pain rather than pediatric dentistry. An additional limitation is that all information was self-reported; thus, providers’ behaviors were not evaluated directly. Last, as the study only focused on pediatric dental providers who were members of the AAPD, the findings may not be applicable to pediatric dentists who are not involved in the AAPD or are located outside the United States.

## Figures and Tables

**Table 1:** Demographic characteristics of the participants.

	Total (N = 206)
Demographics	
Gender	N (%)
Woman	135 (65.5%)
Man	67 (32.5%)
Prefer not to answer	4 (1.9%)
Age	N (%)
Between 25–35 years	61 (29.6%)
Between 36–45 years	61 (29.6%)
Between 46–55 years	44 (21.4%)
Between 56–65 years	21 (10.2%)
Over 66 years	18 (8.7%)
Ethnicity	N (%)
Hispanic or Latinx	11 (5.3%)
Not Hispanic or Latinx	173 (84.0%)
Prefer not to answer	22 (10.7%)
Race	N (%)
White	132 (64.1%)
Black of African American	7 (3.4%)
Asian	42 (20.4%)
Other	7 (3.4%)
Prefer not to answer	18 (8.7%)
Occupation	
Working days per week	N (%)
0 day	4 (1.9%)
1–3 days	38 (18.5%)
4–7 days	164 (79.6%)
Primary clinical setting	N (%)
Private practice	165 (80.1%)
Academic institution	14 (6.8%)
Community clinic	13 (6.3%)
Hospital	10 (4.9%)
Other	4 (1.9%)
Region	N (%)
Northeast	64 (31.1%)
Midwest	34 (16.5%)
West	35 (17.0%)
Southeast	46 (22.3%)
Southwest	27 (13.1%)
Educational Qualifications	
Additional specialty training	N (%)
GPR	34 (16.5%)
AEGD	13 (6.3%)
Orthodontics and Dentofacial Orthopedics	12 (5.8%)
OMFS	3 (1.5%)
OMFP	2 (1.0%)
Oral Medicine	1 (0.5%)
Orofacial Pain	1 (0.5%)
Endodontics	1 (0.5%)
Periodontics	1 (0.5%)
Other	5 (2.4%)
Additional training relevant to TMD	N (%)
Continuing education courses	101 (49.0%)
Attendance at scientific conferences	30 (14.6%)
Postgraduate specialty training	13 (6.3%)
Simulation training	1 (0.5%)
Other	9 (4.4%)
None	90 (43.7%)

Abbreviations: AEGD: Advanced Education in General Dentistry; GPR: General Practice Residency; OMFP: Oral and Maxillofacial Pathology; OMFS: Oral and Maxillofacial Surgery; TMD: temporomandibular disorder. All the data are reported as frequency number and percentage.

**Table 2:** Respondents’ perceived knowledge and confidence levels, and frequency of performing TMD assessments, according to number of years from graduation.

Outcome	Total (N = 206)	<2 Years Post Residency (N = 32)	2–5 Years Post Residency (N = 39)	6–10 Years Post Residency (N = 36)	>10 Years Post Residency (N = 99)	*p* Value (Effect Size)
Perceived TMD Knowledge and Confidence Levels in Pediatric Populations (mean ± SD)						
General knowledge about TMD	43.6 ± 27.0	37.2 ± 23.7	39.3 ± 25.5	44.6 ± 26.5	46.9 ± 28.6	0.226 (0.02)
Screening of TMD	50.3 ± 31.7	49.2 ± 29.0	44.0 ± 30.6	53.5 ± 34.4	52.0 ± 32.1	0.526 (0.01)
Diagnosis of TMD	41.3 ± 29.8	36.5 ± 25.6	35.2 ± 27.2	43.4 ± 29.4	44.5 ± 31.8	0.288 (0.02)
Management of TMD	30.4 ± 27.9	26.0 ± 21.4	28.4 ± 24.9	31.7 ± 29.3	32.2 ± 30.4	0.684 (0.01)
Sufficient training in screening, diagnosis, and management of TMD during pediatric dentistry residency	32.8 ± 27.4	33.9 ± 26.0	28.4 ± 26.8	32.8 ± 26.2	34.1 ± 28.7	0.742 (0.01)
Frequency of Assessments (N, %)						
Screening History						
Difficulty in mouth opening						0.282 (0.15)
Always	36 (17.5%)	1 (3.1%)	6 (15.4%)	5 (13.9%)	24 (24.2%)	
Most of the time	28 (13.6%)	3 (9.4%)	6 (15.4%)	3 (8.3%)	16 (16.2%)	
About half the time	7 (3.4%)	2 (6.3%)	0 (0.0%)	2 (5.6%)	3 (3.0%)	
Sometimes	93 (45.1%)	19 (59.4%)	19 (48.7%)	17 (47.2%)	38 (38.4%)	
Never	42 (20.4%)	7 (21.9%)	8 (20.5%)	9 (25.0%)	18 (18.2%)	
TMJ noise						0.490 (0.14)
Always	32 (15.5%)	1 (3.1%)	6 (15.4%)	5 (13.9%)	20 (20.2%)	
Most of the time	29 (14.1%)	7 (21.9%)	6 (15.4%)	3 (8.3%)	13 (13.1%)	
About half the time	10 (4.9%)	1 (3.1%)	1 (2.6%)	2 (5.6%)	6 (6.1%)	
Sometimes	96 (46.6%)	18 (56.3%)	17 (43.6%)	16 (44.4%)	45 (45.5%)	
Never	39 (18.9%)	5 (15.6%)	9 (23.1%)	10 (27.8%)	15 (15.2%)	
Pain in TMJ or pre-auricular area						0.475 (0.14)
Always	36 (17.5%)	2 (6.3%)	7 (17.9%)	8 (22.2%)	19 (19.2%)	
Most of the time	25 (12.1%)	6 (18.8%)	4 (10.3%)	3 (8.3%)	12 (12.1%)	
About half the time	16 (7.8%)	0 (0.0%)	2 (5.1%)	4 (11.1%)	10 (10.1%)	
Sometimes	83 (40.3%)	17 (53.1%)	15 (38.5%)	12 (33.3%)	39 (39.4%)	
Never	46 (22.3%)	7 (21.9%)	11 (28.2%)	9 (25.0%)	19 (19.2%)	
Pain with jaw function						0.460 (0.14)
Always	38 (18.4%)	3 (9.4%)	9 (23.1%)	5 (13.9%)	21 (21.2%)	
Most of the time	33 (16.0%)	5 (15.6%)	4 (10.3%)	5 (13.9%)	19 (19.2%)	
About half the time	11 (5.3%)	2 (6.3%)	2 (5.1%)	3 (8.3%)	4 (4.0%)	
Sometimes	92 (44.7%)	18 (56.3%)	17 (43.6%)	13 (36.1%)	44 (44.4%)	
Never	32 (15.5%)	4 (12.5%)	7 (17.9%)	10 (27.8%)	11 (11.1%)	
Change in bite						0.442 (0.14)
Always	22 (10.7%)	1 (3.1%)	6 (15.4%)	3 (8.3%)	12 (12.1%)	
Most of the time	20 (9.7%)	3 (9.4%)	5 (12.8%)	4 (11.1%)	8 (8.1%)	
About half the time	11 (5.3%)	3 (9.4%)	3 (7.7%)	1 (2.8%)	4 (4.0%)	
Sometimes	93 (45.1%)	15 (46.9%)	13 (33.3%)	13 (36.1%)	52 (52.5%)	
Never	60 (29.1%)	10 (31.3%)	12 (30.8%)	15 (41.7%)	23 (23.2%)	
Episodes of jaw lock						0.557 (0.13)
Always	24 (11.7%)	1 (3.1%)	5 (12.8%)	3 (8.3%)	15 (15.2%)	
Most of the time	10 (4.9%)	2 (6.3%)	3 (7.7%)	2 (5.6%)	3 (3.0%)	
About half the time	11 (5.3%)	1 (3.1%)	1 (2.6%)	4 (11.1%)	5 (5.1%)	
Sometimes	115 (55.8%)	22 (68.8%)	20 (51.3%)	17 (47.2%)	56 (56.6%)	
Never	46 (22.3%)	6 (18.8%)	10 (25.6%)	10 (27.8%)	20 (20.2%)	
Previous injuries to jaw, head, neck						0.127 (0.17)
Always	50 (24.3%)	6 (18.8%)	7 (17.9%)	11 (30.6%)	26 (26.3%)	
Most of the time	25 (12.1%)	3 (9.4%)	8 (20.5%)	2 (5.6%)	12 (12.1%)	
About half the time	10 (4.9%)	5 (15.6%)	0 (0.0%)	2 (5.6%)	3 (3.0%)	
Sometimes	89 (43.2%)	14 (43.8%)	16 (41.0%)	14 (38.9%)	45 (45.5%)	
Never	32 (15.5%)	4 (12.5%)	8 (20.5%)	7 (19.4%)	13 (13.1%)	
Previous treatment for TMD						0.380 (0.14)
Always	15 (7.3%)	1 (3.1%)	5 (12.8%)	3 (8.3%	6 (6.1%)	
Most of the time	8 (3.9%)	1 (3.1%)	1 (2.6%)	0 (0.0%)	6 (6.1%)	
About half the time	3 (1.5%)	0 (0.0%)	0 (0.0%)	2 (5.6%)	1 (1.0%)	
Sometimes	76 (36.9%)	10 (31.3%)	14 (35.9%)	12 (33.3%)	40 (40.4%)	
Never	104 (50.5%)	20 (62.5%)	19 (48.7%)	19 (52.8%)	46 (46.5%)	
Parafunctional activities						0.137 (0.17)
Always	75 (36.4%)	8 (25.0%)	17 (43.6%)	11 (30.6%)	39 (39.4%)	
Most of the time	44 (21.4%)	13 (40.6%)	6 (15.4%)	10 (27.8%)	15 (15.2%)	
About half the time	36 (17.5%)	6 (18.8%)	9 (23.1%)	7 (19.4%)	14 (14.1%)	
Sometimes	42 (20.4%)	4 (12.5%)	5 (12.8%)	6 (16.7%)	27 (27.3%)	
Never	9 (4.4%)	1 (3.1%)	2 (5.1%)	2 (5.6%)	4 (4.0%)	
Clinical Assessment						
Masticatory muscle palpation						0.281 (0.15)
Always	81 (39.3%)	8 (25.0%)	11 (28.2%)	19 (52.8%)	43 (43.4%)	
Most of the time	36 17.5%)	9 (28.1%)	6 (15.4%)	4 (11.1%)	17 (17.2%)	
About half the time	22 (10.7%)	4 (12.5%)	7 (17.9%)	4 (11.1%)	7 (7.1%)	
Sometimes	45 (21.8%)	9 (28.1%)	9 (23.1%)	5 (13.9%)	22 (22.2%)	
Never	22 (10.7%)	3 (6.3%)	6 (15.4%)	4 (11.1%)	10 (10.1%)	
TMJ palpation						0.057 (0.18)
Always	82 (39.8%)	9 (28.1%)	14 (35.9%)	20 (55.6%)	39 (39.4%)	
Most of the time	42 (20.4%)	8 (25.0%)	5 (12.8%)	6 (16.7%)	23 (23.2%)	
About half the time	18 (8.7%)	5 (15.6%)	7 (17.9%)	3 (8.3%)	3 (3.0%)	
Sometimes	45 (21.8%)	6 (18.8%)	7 (17.9%)	5 (13.9%)	27 (27.3%)	
Never	19 (9.2%)	4 (12.5%)	6 (15.4%)	2 (5.6%)	7 (7.1%)	
TMJ noise auscultation						0.003 * (0.22)
Always	43 (20.9%)	5 (15.6%)	7 (17.9%)	14 (38.9%)	17 (17.2%)	
Most of the time	21 (10.2%)	3 (9.4%)	4 (10.3%)	8 (22.2%)	6 (6.1%)	
About half the time	11 (5.3%)	3 (9.4%)	4 (10.3%)	1 (2.8%)	3 (3.0%)	
Sometimes	37 (18.0%)	9 (28.1%)	3 (7.7%)	2 (5.6%)	23 (23.2%)	
Never	94 (45.6%)	12 (37.5%)	21 (53.8%)	11 (30.6%)	50 (50.5%)	
Mandibular range of motion						0.105 (0.17)
Always	70 (34.0%)	8 (25.0%)	12 (30.8%)	16 (44.4%)	34 (34.3%)	
Most of the time	29 (14.1%)	4 (12.5%)	1 (2.6%)	7 (19.4%)	17 (17.2%)	
About half the time	12 (5.8%)	3 (9.4%)	5 (12.8%)	0 (0.0%)	4 (4.0%)	
Sometimes	52 (25.2%)	7 (21.9%)	10 (25.6%)	8 (22.2%)	27 (27.3%)	
Never	43 (20.9%)	10 (31.3%)	11 (28.2%)	5 (13.9%)	17 (17.2%)	

Abbreviations: TMD: temporomandibular disorder; TMJ: temporomandibular joint. * signifies statistically significant difference.

**Table 3:** Perceived knowledge and confidence levels about TMD in pediatric populations according to frequency of TMD assessment during screening history.

Screening Assessment Item	Frequency of Screening Assessment	General Knowledge about TMD	Screening of TMD	Diagnosis of TMD	Management of TMD	Sufficient Training in TMD during Pediatric Dentistry Residency
Screening History						
Difficulty in mouth opening	Always/Most of the time (N = 64)	60.8 ± 27.9	67.1 ± 28.7	57.2 ± 30.5	46.0 ± 32.0	45.5 ± 32.4
	Sometimes/Never (N = 135)	35.5 ± 23.0	42.0 ± 29.9	33.4 ± 27.0	23.3 ± 22.9	26.6 ± 22.7
	*p* value (effect size)	<0.001 * (1.0)	<0.001 * (0.85)	<0.001 * (0.83)	<0.001 * (0.87)	<0.001 * (0.72)
TMJ noise	Always/Most of the time (N = 39)	46.6 ± 22.6	57.4 ± 27.7	42.6 ± 26.2	30.4 ± 26.1	34.1 ± 24.3
	Sometimes/Never (N = 39)	27.3 ± 20.9	32.2 ± 20.3	25.4 ± 26.2	17.0 ± 19.6	17.8 ± 19.7
	*p* value (effect size)	<0.001 * (0.89)	<0.001 * (0.87)	0.005 * (0.66)	0.012 * (0.58)	0.002 * (0.74)
Pain in TMJ or pre-auricular area	Always/Most of the time (N = 41)	47.4 ± 23.7	54.8 ± 26.2	45.2 ± 26.0	38.0 ± 24.0	37.7 ± 24.9
	Sometimes/Never (N = 46)	27.8 ± 20.9	33.2 ± 29.8	27.4 ± 26.7	18.9 ± 21.2	19.0 ± 19.9
	*p* value (effect size)	<0.001 * (0.88)	<0.001 * (0.77)	0.002 * (0.68)	<0.001 * (0.85)	<0.001 * (0.83)
Pain with jaw function	Always/Most of the time (N = 71)	56.7 ± 29.1	63.1 ± 30.6	53.1 ± 32.3	42.9 ± 32.1	43.7 ± 32.3
	Sometimes/Never (N = 124)	35.8 ± 23.2	42.6 ± 30.4	34.5 ± 26.5	23.1 ± 23.2	26.7 ± 22.7
	*p* value (effect size)	<0.001 * (0.82)	<0.001 * (0.67)	<0.001 * (0.65)	<0.001 * (0.74)	<0.001 * (0.64)
Change in bite	Always/Most of the time (N = 42)	59.6 ± 30.5	67.3 ± 32.1	58.2 ± 33.7	49.3 ± 34.8	49.5 ± 33.7
	Sometimes/Never (N = 153)	39.3 ± 25.0	45.1 ± 30.7	36.0 ± 27.1	25.1 ± 23.9	28.5 ± 2.0
	*p* value (effect size)	<0.001 * (0.73)	<0.001 * (0.72)	<0.001 * (0.77)	<0.001 * (0.91)	<0.001 * (0.79)
Episodes of jaw lock	Always/Most of the time (N = 34)	71.8 ± 26.1	80.1 ± 21.3	69.5 ± 27.4	56.6 ± 31.8	60.9 ± 33.0
	Sometimes/Never (N = 161)	37.3 ± 23.6	43.6 ± 29.3	34.7 ± 26.8	24.2 ± 23.4	27.1 ± 22.5
	*p* value (effect size)	<0.001 * (1.43)	<0.001 * (1.27)	<0.001 * (1.29)	<0.001 * (1.30)	<0.001 * (1.37)
Previous injuries to jaw, head, neck	Always/Most of the time (N = 75)	56.4 ± 28.1	63.8 ± 31.2	53.3 ± 30.9	41.1 ± 31.0	41.7 ± 31.9
	Sometimes/Never (N = 121)	35.8 ± 23.3	41.8 ± 29.2	33.3 ± 25.9	23.0 ± 22.5	27.4 ± 23.1
	*p* value (effect size)	<0.001 * (0.82)	<0.001 * (0.74)	<0.001 * (0.71)	<0.001 * (0.70)	<0.001 * (0.54)
Previous treatment for TMD	Always/Most of the time (N = 23)	72.6 ± 24.1	81.9 ± 23.6	72.3 ± 29.1	59.4 ± 34.3	58.9 ± 32.0
	Sometimes/Never (N = 180)	39.5 ± 25.1	46.2 ± 30.5	37.2 ± 27.6	26.6 ± 24.7	29.0 ± 24.8
	*p* value (effect size)	<0.001 * (1.32)	<0.001 * (1.20)	<0.001 * (1.27)	<0.001 * (1.27)	<0.001 * (1.16)
Parafunctional activities	Always/Most of the time (N = 119)	50.2 ± 27.7	60.1 ± 30.7	48.1 ± 30.6	35.3 ± 30.1	27.5 ± 30.0
	Sometimes/Never (N = 51)	34.5 ± 24.6	35.4 ± 28.9	33.5 ± 29.1	23.8 ± 24.2	25.7 ± 22.1
	*p* value (effect size)	<0.001 * (0.59)	<0.001 * (0.82)	0.004* (0.48)	0.010 * (0.40)	0.005 * (0.42)
Clinical Assessment						
Masticatory muscle palpation	Always/Most of the time (N = 117)	53.9 ± 26.7	62.5 ± 29.9	51.6 ± 30.7	39.2 ± 29.4	40.6 ± 29.3
	Sometimes/Never (N = 67)	28.0 ± 21.0	30.6 ± 25.8	25.6 ± 22.7	17.8 ± 21.8	20.4 ± 20.7
	*p* value (effect size)	<0.001 * (1.05)	<0.001 * (1.12)	<0.001 * (0.93)	<0.001 * (0.79)	<0.001 * (0.76)
TMJ palpation	Always/Most of the time (N = 124)	51.1 ± 27.3	58.9 ± 31.7	48.7 ± 31.2	36.9 ± 29.6	38.2 ± 29.4
	Sometimes/Never (N = 64)	30.9 ± 22.4	33.9 ± 26.5	28.8 ± 23.9	19.8 ± 22.7	23.0 ± 21.9
	*p* value (effect size)	<0.001 * (0.78)	<0.001 * (0.83)	<0.001 * (0.69)	<0.001 * (0.62)	<0.001 * (0.56)
TMJ noise auscultation	Always/Most of the time (N = 64)	54.1 ± 25.6	64.2 ± 29.4	54.7 ± 30.2	39.5 ± 30.7	42.6 ± 28.6
	Sometimes/Never (N = 131)	38.0 ± 26.1	42.5 ± 30.6	34.6 ± 27.6	25.7 ± 25.4	27.3 ± 25.0
	*p* value (effect size)	<0.001 * (0.62)	<0.001 * (0.72)	<0.001 * (0.71)	0.002 * (0.51)	<0.001 * (0.58)
Mandibular range of motion	Always/Most of the time (N = 99)	56.1 ± 26.1	64.6 ± 28.4	53.6 ± 29.7	40.7 ± 29.1	42.6 ± 29.2
	Sometimes/Never (N = 95)	31.1 ± 22.1	34.7 ± 28.1	29.0 ± 24.6	20.0 ± 22.4	22.7 ± 21.5
	*p* value (effect size)	<0.001 * (1.03)	<0.001 * (1.06)	<0.001 * (0.91)	<0.001 * (0.80)	<0.001 * (0.78)

Abbreviations: TMD: temporomandibular disorder; TMJ: temporomandibular joint. * signifies statistically significant difference.

## Data Availability

The original contributions presented in the study are included in the article/Supplementary Materials; further inquiries can be directed to the corresponding author.

## Supplementary Materials

The following supporting information can be downloaded at: https://www.mdpi.com/article/10.3390/children11101168/s1, Survey used in the current study.
