# Supplementary material for: Pediatric Dentists’ Practice Patterns in the Screening, Diagnosis, and Management of Temporomandibular Disorders
Source: Children (Basel). 2024 Sep 26;11(10):1168. doi: 10.3390/children11101168 (PMC11505712; doi:10.3390/children11101168)
Supplement: Supplementary file 1 [file children-11-01168-s001.zip › children-3227076-supplementary.pdf]

Start of Block: Default Question Block

Practice Patterns Among Pediatric Dentists for the Management of Temporomandibular Disorders

Thank you for your interest in this project! The purpose of this survey is to evaluate practice patterns among pediatric dentists in the screening, diagnosis, and management of temporomandibular disorders (TMD) in pediatric patients.

Page Break

UNC
SCHOOL OF DENTISTRY

Practice Patterns Among Pediatric Dentists for the Management of Temporomandibular Disorders

The purpose of this research study is to evaluate practice patterns among pediatric dentists in the screening, diagnosis, and management of temporomandibular disorders (TMD). You are being asked to take part in a research study because you are a pediatric dentist.

Being in a research study is completely voluntary. You can choose not to be in this research study. You can also say yes now and change your mind later.

If you agree to take part in this research, you will be asked to answer questions regarding your clinical practice patterns for managing pediatric patients with signs and/or symptoms of TMD; answer basic demographic questions about you and your training. Your participation in this study will take about 10 minutes. We expect that all pediatric dentist members of the American Academy of Pediatric Dentistry (9,800 people) will take part in this research study.

You can choose not to answer any question you do not wish to answer. You can also choose to stop taking the survey at any time. You must be at least 18 years old to participate. If you are younger than 18 years old, please stop now.

The possible risks to you in taking part in this research are potential loss of confidentiality of data.

To protect your identity as a research subject, no identifiable information will be collected.

If you have any questions about this research, please contact the Investigator named at the top of this form by calling 919-537-3200 or emailing caroline_sawicki@unc.edu. If you have questions or concerns about your rights as a research subject, you may contact the UNC Institutional Review Board at 919-966-3113 or by email to IRB_subjects@unc.edu.

By proceeding with the survey, you are consenting to participate in the study.

Page Break

First, please answer a question to ensure that you're eligible.

Page Break

Are you currently completing or have already completed specialized training in pediatric dentistry from a CODA-approved program in the United States?

- ◯ Yes
- ◯ No

Skip To: End of Survey If Are you currently completing or have already completed specialized training in pediatric dentistr... = No

Page Break

Great- you're eligible to complete the survey!

Page Break

UNC SCHOOL OF DENTISTRY

Practice Patterns Among Pediatric Dentists for the Management of Temporomandibular Disorders

Please note:

- This survey takes about 10 minutes to complete.
- If you start the survey and then get interrupted, you can go back to the link and finish it at a later time.
- Your answers are anonymous and confidential.
- This survey is voluntary and you may choose not to participate.
- 25 $100 Amazon e-gift cards will be raffled off to participants who choose to provide their email addresses at the end of the survey. If you choose to participate in the raffle, your responses will still remain anonymous.

If you have any questions or issues with the survey, such as it freezes or you get kicked out, please email us: **caroline_sawicki@unc.edu.**

Thank you for your time,
Caroline Sawicki, DDS, PhD and Linda Sangalli, DDS, PhD, MS

Page Break

UNC SCHOOL OF DENTISTRY

For the first set of questions, please state your level of agreement with the following statements.

Page Break

UNC SCHOOL OF DENTISTRY

Q1
Please state your agreement level (0=strongly disagree, 100=strongly agree).

	0 10 20 30 40 50 60 70 80 90 100
I am knowledgable about TMD in pediatric populations	
I received sufficient training in the screening, diagnosis, and management of TMD during my pediatric dentistry residency	
I feel comfortable screening for TMD in pediatric patients	
I feel comfortable diagnosing TMD in pediatric patients	
I feel comfortable managing TMD in pediatric patients	

Page Break

For the next set of questions, please indicate the frequency of specific questions you ask and clinical examination items you use in practice.

Page Break

Q2 How often do you ask pediatric patients (and/or their parent) *“Do you have difficulty opening your mouth?”*

- ◯ Always
- ◯ Most of the time
- ◯ About half the time
- ◯ Sometimes
- ◯ Never

Q3 How often do you ask pediatric patients (and/or their parent) *“Do you hear noises within your jaw joint or in front of your ears?”*

- ◯ Always
- ◯ Most of the time
- ◯ About half the time
- ◯ Sometimes
- ◯ Never

Q4 How often do you ask pediatric patients (and/or their parent) *"Do you have pain in or around your ears or your cheeks?"*

- Always
- Most of the time
- About half the time
- Sometimes
- Never

Q5 How often do you ask pediatric patients (and/or their parent) *"Do you have pain when chewing, talking, opening wide, or using your jaw?"*

- Always
- Most of the time
- About half the time
- Sometimes
- Never

Q6 How often do you ask pediatric patients (and/or their parent) *"Has your bite felt uncomfortable or unusual?"*

- Always
- Most of the time
- About half the time
- Sometimes
- Never

Q7 How often do you ask pediatric patients (and/or their parent) *"Does your jaw ever lock or go out of place?"*

- Always
- Most of the time
- About half the time
- Sometimes
- Never

Q8 How often do you ask pediatric patients (and/or their parent) *“Have you ever had an injury to your jaw, head, or neck?”*

- Always
- Most of the time
- About half the time
- Sometimes
- Never

Q9 How often do you ask pediatric patients (and/or their parent) *“Have you previously been treated for temporomandibular disorders?"*

- Always
- Most of the time
- About half the time
- Sometimes
- Never

Q10 How often do you ask pediatric patients (and/or their parent) *"Do you grind or clench your teeth during the day or at night?"*

- Always
- Most of the time
- About half the time
- Sometimes
- Never

Q11 During clinical exam, how often do you palpate the muscles of mastication for tenderness, pain, or pain referral patterns in pediatric patients?

- Always
- Most of the time
- About half the time
- Sometimes
- Never

UNC SCHOOL OF DENTISTRY

Q12 During clinical exam, how often do you palpate the TMJ for pain and/or tenderness in pediatric patients?

- Always
- Most of the time
- About half the time
- Sometimes
- Never

Q13 During clinical exam, how often do you auscultate for TMJ sounds in pediatric patients?

- Always
- Most of the time
- About half the time
- Sometimes
- Never

Q14 During clinical exam, how often do you assess for mandibular range of motion in pediatric patients?

- Always
- Most of the time
- About half the time
- Sometimes
- Never

Page Break

For the next set of questions, please indicate your current clinical encounters.

Page Break

Q15 On average, what percentage of pediatric patients do you see presenting with TMD signs and/or symptoms per week?

- ○ 0%
- ○ Up to 25%
- ○ Up to 50%
- ○ Up to 75%
- ○ Almost 100%
- ○ I don’t routinely assess for TMD signs and/or symptoms in pediatric patients.

Q16 What is the predominant age group that you see presenting with signs and/or symptoms of TMD? *Check all that apply.*

- ▢ 5 years or younger
- ▢ 6 to 8 years
- ▢ 9 to 11 years
- ▢ 12 to 14 years
- ▢ 15 to 17 years
- ▢ 18 years or older
- ▢ None of my pediatric patients present with signs and/or symptoms of TMD.
- ▢ I don't routinely assess for TMD signs and/or symptoms in pediatric patients.

Q17 When you have a patient presenting with signs and/or symptoms of TMD, what treatment do you recommend? *Check all that apply.*

- ▢ Fabricate occlusal splint
- ▢ Perform occlusal adjustment
- ▢ Refer to specialist
- ▢ Patient education
- ▢ Physical therapy (e.g., jaw exercises, massage)
- ▢ Behavioral therapy (e.g., biofeedback, relaxation training, stress management, etc.)
- ▢ Prescription medication (e.g., non-steroidal anti-inflammatory drugs, anxiolytic agents, muscle relaxants, etc.)
- ▢ Order TMJ imaging assessment (e.g., panoramic radiograph, computed tomography, magnetic resonance imaging, etc.)
- ▢ None of my pediatric patients present with signs and/or symptoms of TMD.
- ▢ I don't routinely assess for TMD signs and/or symptoms in pediatric patients.

Display This Question:

If When you have a patient presenting with signs and/or symptoms of TMD, what treatment do you recom... = Refer to specialist

Q18 Which specialist(s) do you most often refer to when a pediatric patient presents with signs and/or symptoms with TMD? *Check all that apply.*

- ☐ Orthodontist
- ☐ Orofacial pain specialist
- ☐ Oral and maxillofacial surgeon
- ☐ Primary care physician
- ☐ Psychologist
- ☐ Physical therapist
- ☐ Other; please describe ____

Q19 Which co-morbidities do you most commonly encounter in pediatric patients presenting with TMD signs and/or symptoms? *Check all that apply.*

- ☐ Sleep disorders
- ☐ Arthritis
- ☐ Irritable bowel syndrome
- ☐ Headache/migraine
- ☐ Allergies
- ☐ Anxiety
- ☐ Depression
- ☐ Behavioral/developmental disorders (e.g., autism spectrum disorder, attention-deficit/hyperactivity disorder)
- ☐ Other; please describe ____
- ☐ None of my pediatric patients present with signs and/or symptoms of TMD.
- ☐ I don't routinely assess for TMD signs and/or symptoms in pediatric patients.

Page Break

UNC SCHOOL OF DENTISTRY

These last few questions ask about you and your training.

Page Break

UNC SCHOOL OF DENTISTRY

Q20 Besides pediatric dentistry residency, what additional training have you received that is specifically relevant to managing TMD in pediatric patients?

- ☐ Additional postgraduate specialty training
- ☐ Continuing education courses
- ☐ Simulation training
- ☐ Attendance at national and/or international conferences
- ☐ Other; please describe ____
- ☐ None

Q21 Which of these educational resources would be most helpful for you as a pediatric dentist working with pediatric patients presenting with TMD signs and/or symptoms? *Check all that apply.*

- ☐ More studies to determine the prevalence of TMD and its clinical implications for practice
- ☐ Continuing education courses and training focused on the management of TMD
- ☐ Clearer guidance from national societies for the screening, diagnosis, and management of TMD
- ☐ Other; please describe ____

UNC SCHOOL OF DENTISTRY

Q22 How many years have you been practicing pediatric dentistry?

- ◯ Currently in pediatric dentistry residency
- ◯ 0-2 years post residency
- ◯ 2-5 years post residency
- ◯ 5-10 years post residency
- ◯ 10+ years post residency

Q23 How many days per week do you practice or encounter pediatric dentistry?

- ◯ 0
- ◯ 1
- ◯ 2
- ◯ 3
- ◯ 4
- ◯ 5
- ◯ 6+

Q24 What type of clinical setting do you primarily work in?

- ○ Community clinic or federally qualified health center
- ○ Private practice
- ○ Hospital
- ○ Academic institution
- ○ Other; please describe ____

Q25 Which region do you primarily practice in?

- ○ Northeast
- ○ Midwest
- ○ West
- ○ Southeast
- ○ Southwest

UNC SCHOOL OF DENTISTRY

Q26 In addition to pediatric dentistry, what other dental training have you received? *Check all that apply.*

- ☐ General Practice Residency
- ☐ Advanced Education in General Dentistry
- ☐ Oral Medicine
- ☐ Orofacial Pain
- ☐ Dental Anesthesiology
- ☐ Oral and Maxillofacial Surgery
- ☐ Oral and Maxillofacial Pathology
- ☐ Orthodontics
- ☐ Endodontics
- ☐ Prosthodontics
- ☐ Periodontics
- ☐ Dental Public Health
- ☐ Other; please describe ____
- ☐ None

UNC SCHOOL OF DENTISTRY

Q27 What is your age?

____

Q28 Which gender do you identify with?

- ○ Male
- ○ Female
- ○ Non-binary / third gender
- ○ Prefer not to answer

Q29 Which ethnicity do you identify with?

- ○ Hispanic or Latinx
- ○ Not Hispanic or Latinx
- ○ Unsure
- ○ Prefer not to answer

Q30 What race do you identify with?

- White
- Black or African American
- American Indian or Alaska Native
- Asian
- Native Hawaiian or Other Pacific Islander
- Other
- Unsure
- Prefer not to answer

Page Break

Please share any other thoughts or feedback related to managing TMD in pediatric dental patients from a pediatric dentist's perspective.

____

Page Break

End of Block: Default Question Block

Start of Block: Block 1

Would you like to be entered into a raffle to win one of 25 $100 Amazon e-gift cards? Your responses will still remain anonymous.

- ○ Yes
- ○ No

End of Block: Block 1
